# Immunogenicity of one dose and two doses of adjuvanted influenza vaccine in lung transplant candidates

**DOI:** 10.1371/journal.pone.0332346

**Published:** 2025-09-18

**Authors:** Valentina Polishchuk, Мikhail Kostinov, Аleksey Ryzhov, Yulia Dagil, Izabella Khrapunova, Alexander Zhestkov, Evgeny Tarabrin, Natalia Karchevskaya, Anna Vlasenko, Aristitsa Kostinova, Arseniy Poddubikov, Irina Solovieva, Anna Khamidulina, Ekaterina Prutskova, Irina Mekhantseva

**Affiliations:** 1 Laboratory for Vaccination and Immunotherapy of Allergic Diseases, I. I. Mechnikov Research Institute of Vaccines and Sera, Moscow, Russian Federation; 2 Department of Epidemiology and Modern Vaccination Technology, I.M. Sechenov First Moscow State Medical University, Moscow, Russian Federation; 3 Laboratory of Clinical Immunology, National Research Center — Institute of Immunology, Federal Medical and Biological Agency of Russia, Moscow, Russian Federation; 4 Department of Clinical Medicine, Medical University “Reaviz”, Samara, Russian Federation; 5 Department of Hospital Surgery, I.M. Sechenov First Moscow State Medical University, Moscow, Russian Federation; 6 Department of Thoracic and Abdominal Surgery, N.V. Sklifosovsky Research Institute for Emergency Medicine, Moscow, Russian Federation; 7 Center for Evidence-Based Medicine and Biostatistics, Samara State Medical University, Samara, Russian Federation; 8 N.V. Sklifosovsky Institute of Clinical Medicine, I. M. Sechenov First Moscow State Medical University (Sechenov University), Moscow, Russia; 9 Department of Pediatrics, Ulyanovsk State University, Ulyanovsk, Russian Federation; 10 Department of Internal Medicine, Karaganda Medical University, Karaganda, Kazakhstan; IAVI, UNITED STATES OF AMERICA

## Abstract

Influenza is especially dangerous for high-risk patients, for whom various vaccination strategies are used to prevent this disease. This study assessed the safety and immunological effectiveness of an inactivated trivalent polymer-subunit influenza vaccine Grippol® Plus administered according to two regimens in a group of 41 patients with severe progressive bronchopulmonary disorders. It also investigated the duration of vaccine-induced antibody response in this patient population. Group 1 of the study subjects (n = 21) received one dose of Grippol® Plus, and Group 2 (n = 20) received two doses of Grippol® Plus one month apart in the same influenza season. To measure antibody levels, paired sera were tested in hemagglutination inhibition assay pre-vaccination and at months 1 and 12 post-vaccination. Vaccine immunogenicity was assessed by the Committee for Medicinal Products for Human Use (CHMP) criteria. All patients were symptom-free in the post-vaccination period. The one-dose regimen met all CHMP criteria against strains A/California/7/2009(H1N1)pdm09-like and A/Texas/50/2012(H3N2) and the criteria for seroprotection and seroconversion against the B/Massachusetts/2/2012 strain. This regimen was associated with a significant increase in Ab titers against all the three influenza viral strains. In the two-dose group, no statistically significant differences were observed in IgG Ab levels, seroprotection rate, seroconversion rate or seroconversion factor at month 1 after the first and second vaccine doses. At month 12 post-vaccination, Ab levels returned to baseline, regardless the vaccination regimen. In lung transplant candidates, one-dose vaccination with an inactivated trivalent polymer-subunit influenza vaccine was effective in generating protective antibody levels for the current influenza season.

## Introduction

Influenza is a very important healthcare issue. It is associated with a higher risk of hospital admission and death both in immunocompetent and immunocompromised patients, including patients with severe progressive bronchopulmonary disorders [1]. In this patient population, influenza evolves as a more severe disease and increases the risk of bacterial and viral superinfection, which could be fatal for patients with severe respiratory failure. Thus, patients with bronchopulmonary disorders should receive annual vaccinations against influenza [2–4]. They reduce the frequency of exacerbations of the underlying disease and the rate of hospital admissions [5–8]. R. Garrastazu et al. showed that positive effects of influenza vaccination were more pronounced in patients with severe chronic obstructive pulmonary disease (COPD) [5].

Despite the national and international recommendations, even for high-risk individuals influenza vaccination coverage remains low [9]. Among COPD patients, the percentage of vaccinated individuals ranges from 11.3 to 63%, and among solid organ transplant recipients from 48 to 52% [5,10–13]. A. Mohr et al. assessed data on vaccination coverage for seasonal influenza collected during several years and showed that only 11.3–18% of COPD patients and 12.5–26% of patients with interstitial lung disease had been immunized [10].

It is necessary to remember that in patients with severe progressive bronchopulmonary disorders, including lung transplant candidates, influenza vaccination could be less effective due to ongoing treatment, which may include glucocorticoids and sometimes immunosuppressive therapy. Some authors reported that patients with grade 4 COPD have lower titers of vaccine-induced anti-influenza antibodies than patients with grades 1, 2 and 3 COPD [14].

A number of strategies are being discussed to improve the immunogenicity of influenza vaccines in these patient populations, including increasing vaccine doses, using alternative vaccine regimens and adjuvanted vaccines as well as administering vaccines intradermally. However, no clear evidence has been reported supporting the superiority of any of these strategies [15].

### Study objective

To assess the safety and immunological effectiveness of one and two doses of an adjuvanted influenza vaccine in lung transplant candidates.

## Materials and methods

### Patients

Overall, 41 lung transplant candidates with various bronchopulmonary disorders were included in this study aiming to evaluate vaccine-induced influenza immunity. The study was conducted from 29.09.2014 to 30.11.2015. All patients received one dose of an inactivated trivalent polymer-subunit influenza vaccine. One month later, the first 20 of these patients (49%) received a second dose of the vaccine. The study subjects were divided into two groups. Patients in Group 1 (51%; 21/41) received one vaccine dose, and patients in Group 2 (49%; 20/41) received two vaccine doses one month apart.

The study participants with obstructive pulmonary diseases and cystic fibrosis received bronchodilators and mucolytic agents (dornase alfa, sodium chloride 7%, sodium hyaluronate 0.1%, ambroxol hydrochloride, etc.). The patients with cystic fibrosis additionally received antibacterial agents, depending on pathogen susceptibility (cefepime, meropenem, imipenem/cilastatin, linezolid, doripenem, ciprofloxacin, polymyxin and others). The patients with idiopathic pulmonary fibrosis received antifibrotic agents (pirfenidone or nintedanib), and the patients with fibrosis caused by extrinsic allergic alveolitis constantly received small doses of systemic glucocorticoids (SGCs) (10 mg per day).

All study subjects gave written informed consent for inclusion in the study.

The study was conducted in compliance with the guidelines of the Declaration of Helsinki and was approved by the Ethics Committee of the Federal State Budgetary Scientific Institution I.I. Mechnikov Research Institute of Vaccines and Sera.

#### Inclusion criteria.

The study included patients with severe bronchopulmonary disorders who were placed on the lung transplant waiting list, based on their screening results. All the patients gave voluntary consent to receive vaccination and signed a consent form.

#### Non-inclusion criteria.

A patient’s refusal to participate in the study.

### Vaccine

The study vaccine was Grippol® Plus, an inactivated trivalent polymer-subunit 2014–2015 influenza vaccine (Petrovax Pharm, Russia), containing 5 mcg of hemagglutinins of the epidemiologically relevant strains of influenza virus subtypes А(H1N1), А(H3N2) and type B and 500 mcg of azoximer bromide immune adjuvant in one immunization dose. In the Russian Federation, this vaccine has been used in clinical practice for over 20 years to immunize children 6 months of age and older, pregnant women, adults, people older 65 years, and high-risk patients [16]. In accordance with the World Health Organization for the 2014–2015 influenza season, this vaccine contained the A/California/7/2009(H1N1)pdm09-like, A/Texas/50/2012(H3N2) and B/Massachusetts/2/2012 stains.

The influenza vaccine was administered if the patients did not have any signs of respiratory infection or exacerbation of the underlying disease. It was administered intramuscularly in the upper third of arm.

### Sampling

In the one-dose group, blood samples for Ab titers were collected pre-vaccination and at week 4 and month 12 post-vaccination. In the two-dose group, blood samples were taken prior to the administration of the first and second doses, at week 4 after the second dose and at month 12 after the first dose.

### Determination of Ab titers to influenza viral strains

Serum antibody titers to the influenza viral strains A/California/7/2009(H1N1)pdm09-like, A/Texas/50/2012(H3N2) and B/Massachusetts/2/2012 were measured in paired sera by standard hemagglutination inhibition assay using standardized diagnosticums (LLC Diagnostic Products Manufacturing Enterprise) pre-vaccination and at months 1, 2 (in the 2-dose group) and 12 post-vaccination. A 1:40 titer was arbitrarily considered a protective titer.

### Assessing vaccine immunogenicity

In this population of patients with bronchopulmonary disorders, the immunological effectiveness of influenza vaccination was assessed using the following immunogenicity criteria set out by the CHMP and Guidelines 3.1.3490–17 “Study of population immunity to influenza among the population of the Russian Federation”, which are applied for the assessment of vaccine effectiveness in adults 18–60 years: seroprotection level >70%, seroconversion level >40%, and seroconversion factor (n-fold rise in Ab titer) > 2.5 [17,18].

### Statistical analysis

Descriptive statistics for antibody titers was presented as geometric mean titers (GMT) and their 95% confidence interval (CI). Seroconversion factor was determined as geometric mean fold rise from baseline in Ab titers.

Changes over time in Ab levels and inter-group comparisons were done using the linear mixed-effects model (LMEM). The statistical significance of the model coefficients was determined using the Satterthwaite’s approximation for degrees of freedom [19].

Post-hoc comparisons were performed using corresponding contrasts in the calculated model with a Benjamini-Yekutieli correction; these tests were done using the emmeans package [20]. All calculations were done on log-transformed values of Ab titers.

Descriptive statistics for quantitative variables included median and interquartile range, Me [Q1; Q3]. The quantitative variables were compared in the study groups using the Mann-Whitney test.

Absolute and relative (%) frequencies were calculated for qualitative variables, and 95% CI were calculated for seroprotection and seroconversion rates. Contingency tables were used to compare nominal qualitative variables in the study groups using the chi-square test or Fisher’s test (with the expected rates of <5%).

The immunogenicity parameters were calculated and presented as follows:

- Seroconversion factor (n-fold rise in Ab titer) – taking into account a logarithmic distribution, all the calculations were performed on log2 values. Descriptive statistics was presented as geometric mean rates (GMRs) and their 95% confidence interval (95% CI).
- Seroprotection level (the proportion of subjects with titer of >40): descriptive statistics was presented as percentages and their confidence intervals estimated using the Clopper-Pearson method, number of subjects with the specified characteristics (k) and group size (N).
- Seroconversion level (the proportion of subjects with a ≥ 4-fold increase in titer or the switch from seronegative to seropositive status): descriptive statistics was presented as percentages and their confidence intervals estimated using the Clopper-Pearson method, number of subjects with the specified characteristics (k) and group size (N).

The level of statistically significant differences was defined as p ≤ 0.05. All calculations were done using statistical programming environment R (v.3.6, license GNU GPL2).

## Results

The mean age of the study participants was 34 ± 11.7 years. Among them, there were 46,3% (19/41) males and 53,7% females (22/41). Overall, 5% of the patients (2/41) had pulmonary hypertension, 27% (11/41) had cystic fibrosis, 29% (12/41) had obstructive pulmonary diseases and 39% (16/41) suffered from restrictive pulmonary diseases. A total of 39% (16/41) of the patients had a history of glucocorticoid therapy. Twenty-nine percent of the study subjects (11/41) had been vaccinated against influenza in the previous season.

The study groups were comparable in terms of age, gender, type of disease, number of patients with a history of glucocorticoid therapy, number of newly and previously vaccinated individuals, and baseline Ab titers against the A/Texas/50/2012 (H3N2) and B/Massachusetts/2/2012 strains (Table 1).

**Table 1:** Characteristics of the study groups.

Parameter	Group		p value^1^
	Group 1 (n = 21)	Group 2 (n = 20)	
**Vaccination regimen (Group 1: one dose, Group 2: two doses one month apart)**			
**Age**	36 (24; 43)	32 (26; 40)	p = 0.9
**Gender (male)**	57% (12/21)	35% (7/20)	p = 0.27
**Pulmonary hypertension**	10% (2/21)	0% (0/20)	p = 0.29
**Cystic fibrosis**	24% (5/21)	30% (6/20)	
**Obstructive pulmonary diseases**	33% (7/21)	25% (5/20)	
**Restrictive pulmonary diseases**	33% (7/21)	45% (9/20)	
**Glucocorticoid therapy**	33% (7/21)	45% (9/20)	p = 0.66
**Repeat vaccination (previously vaccinated)**	29% (6/21)	25% (5/20)	p = 0.92
**Ab titer against the A/California/7/2009 (H1N1)pdm09-like strain**	24 [14-41]	53 [28-100]	**p = 0.04**
**Ab titer against the A/Texas/50/2012 (H3N2) strain**	30 [17-52]	36 [18-74]	p = 0.73
**Ab titer against the B/Massachusetts/2/2012 strain**	49 [30-80]	35 [21-57]	p = 0.18

The table shows relative and absolute frequencies for categorical variables, median and interquartile ranges for quantitative variables, and geometric mean titers and their 95% confidence intervals for Ab titers. ^1^ The categorical variables were analyzed using the chi-square test or Fisher’s test (with the expected rates of <5%). The quantitative variables were analyzed using the Mann-Whitney test.

Evaluation of Ab levels before seasonal influenza vaccination in a group of 41 lung transplant candidates showed that at baseline protective Ab titers (≥1:40) against the A/California/7/2009(H1N1)pdm09-like, A/Texas/50/2012(H3N2) and B/Massachusetts/2/2012 strains were present in 53.7 [37.4–69.3]% (22/41), 46.3 [30.7–62.6]% (19/41) and 58.5 [42.1–73.7]% (24/41) of the patients. A total of 20 [8.8–34.9]% of the patients (8/41) had protective Ab titers against all the three strains.

At month 1 post-vaccination, a statistically significant rise from baseline was observed in Ab titers against all the three influenza strains (Table 2). The fold increases in Ab titers were 3.8 for the A/California/7/2009(H1N1)pdm09-like strain, 2.9 for the A/Texas/50/2012(H3N2) strain, and 2.1 for the B/Massachusetts/2/2012 strain. The seroprotection and seroconvertion levels met the criteria of immunogenicity for all three influenza strains (Table 2).

**Table 2:** Parameters of immunological effectiveness of influenza vaccination and dynamics of Ab titers in lung transplant candidates one month after vaccination.

Influenza viral strain	Parameters of immunological effectiveness of influenza vaccination			GMT		
	Seroprotection level (%)	Seroconversion factor	Seroconversion level (%)	Pre-vaccination	At month 1	Change over time
**A/California/7/2009** **(H1N1)pdm09-like**	93 [80-98]	3.8	53 [36-68]	36 [24-54]	136 [97-193]	р < 0.001
**A/Texas/50/2012** **(H3N2)**	85 [70-94]	2.9	45 [29-62]	33 [21-50]	97 [67-143]	р = 0.02
**B/Massachusetts** **/2/2012**	93 [80-98]	2.1	45 [29-62]	41 [30-58]	86 [65-113]	р = 0.05

^1^The seroprotection and seroconversion levels were analyzed using the chi-square test or Fisher’s test (with the expected rates of <5%).

Since this study aimed at identifying the most effective influenza vaccination regimen for this patient cohort, a second dose of influenza vaccine was administered to 20 patients in a non-randomized manner after blood sampling at month 1.

Table 3 shows a comparison in immunogenicity parameters between the one- and two-dose vaccination regimens in lung transplant candidates one month after vaccination.

**Table 3:** Parameters of immunological effectiveness of influenza vaccination in lung transplant candidates one month after vaccination by vaccination regimen.

Parameter		Parameters of immunological effectiveness of influenza vaccination		
		Seroprotection level %	Seroconversion factor GMR [95 CI]	Seroconversion level %
**A/California/7/2009(H1N1)pdm09-like**				
**Vaccination regimen**	**One-dose**	90 [68-99]% (18/20)	5.9 [3-11.4]	70 [46-88]% (14/20)
	Two-dose*	93 [68-100]% (14/15)	2.5 [1.4-4.4]	40 [16-68]% (6/15)
	**p value**	p = 1.00	p = 0.08	p = 0.08
**A/Texas/50/2012(H3N2)**				
**Vaccination regimen**	**One-dose**	90 [68-99]% (18/20)	4.1 [2.5-6.7]	60 [36-81]% (12/20)
	Two-dose*	80 [52-96]% (12/15)	2.2 [1.1-4.3]	33 [12-62]% (5/15)
	**p value**	p = 0.63	p = 0.10	p = 0.12
**B/Massachusetts/2/2012**				
**Vaccination regimen**	**One-dose**	100 [83-100]% (20/20)	2.4 [1.5-3.7]	45 [23-68]% (9/20)
	Two-dose*	87 [60-98]% (13/15)	1.6 [1-2.5]	40 [16-68]% (6/15)
	**p value**	p = 0.18	p = 0.50	p = 0.77

The seroprotection and seroconversion levels were analyzed using the chi-square test or Fisher’s test (with the expected rates of < 5%). The seroconversion factor was analyzed using the Mann-Whitney test.

* − 1 month after administration of the second dose of vaccine.

The analysis of immunogenicity of influenza vaccine administered as one dose or two doses given one month apart in patients with severe bronchopulmonary disorders did not reveal any statistically significant correlations between vaccine immunogenicity and administration regimen.

**Fig 1:**
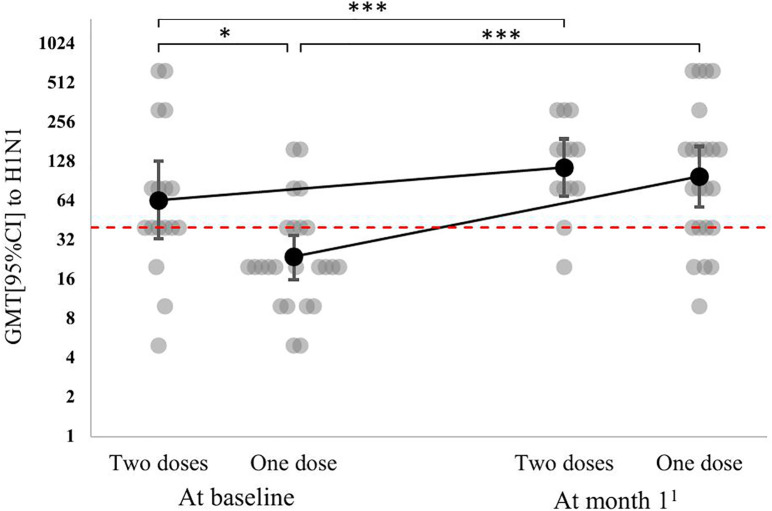
Dynamics of antibody titers to the A/California/7/2009(H1N1)pdm09-like strain by vaccination regimen in lung transplant candidates (individual values and GMTs [95% CI]). * – p < 0.05, *** – p < 0.001 for post-hoc comparisons performed using the corresponding univariant models with a Benjamini-Yekutieli correction. ^1^ – at month 1 after the first dose in the one-dose group and at month 2 after the first dose in the two-dose group. The red broken line shows the minimal protective titer (1:40).

A more detailed analysis of Ab titers to different influenza viral strains in patients who received one and two doses of the study adjuvanted influenza vaccine showed that GMT to the A/California/7/2009(H1N1)pdm09-like strain increased significantly compared with baseline: from 24 [14–41] to 130 [77–220] in the one-dose group (p < 0.001) and from 53 [28–100] to 121 [79–186] in the two-dose group (p = 0.71 for comparison to the titers at month 1 after the administration of the first dose; p < 0.001 for comparison to the baseline value). GMT to the A/California/7/2009(H1N1)pdm09-like strain depended on the vaccination regimen (p = 0.02), the dynamics of GMT also differed depending on the vaccination regimen (p = 0.06 — on the border of statistical significance) (Fig 1). At baseline, GMT to the A/California/7/2009(H1N1)pdm09-like strain was higher in the two-dose group (p = 0.05). At month 1 post-vaccination, GMTs to the A/California/7/2009(H1N1)pdm09-like strain were similar in the one-dose and two-dose groups (p = 0.85).

**Fig 2:**
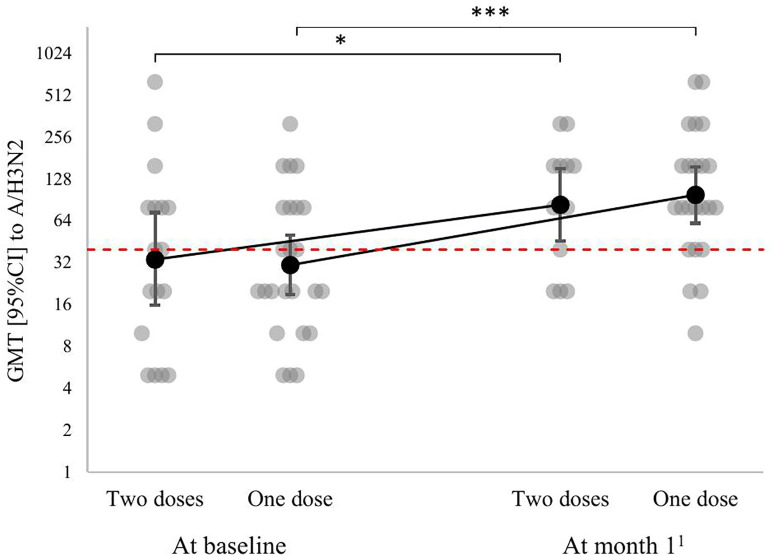
Dynamics of antibody titers to the A/Texas/50/2012(H3N2) strain by vaccination regimen in lung transplant candidates (individual values and GMT [95% CI]). * – p < 0.05, *** – p < 0.001 for post-hoc comparisons performed using the corresponding univariant models with a Benjamini-Yekutieli correction. ^1^ – at month 1 after the first dose in the one-dose group and at month 2 after the first dose in the two-dose group. The red broken line shows the minimal protective titer (1:40).

The antibody titers to the A/Texas/50/2012(H3N2) strain depended only on the fact of vaccination (p < 0.001), and the vaccination regimen did not have a significant effect on the level of antibodies (p < 0.32). Similarly, the dynamics of Ab to the A/Texas/50/2012(H3N2) strain did not depend on the vaccination regimen (p = 0.12) (Fig 2). At month 1, there was a rise in GMT from 30 [17–52] to 117 [70–195] in the one-dose group (p < 0.001) and from 36 [18–74] to 84 [49–143] in the two-dose group (p = 0.82 for comparison to the titers at month 1 after the first dose; p < 0.002 for comparison to the baseline value).

**Fig 3:**
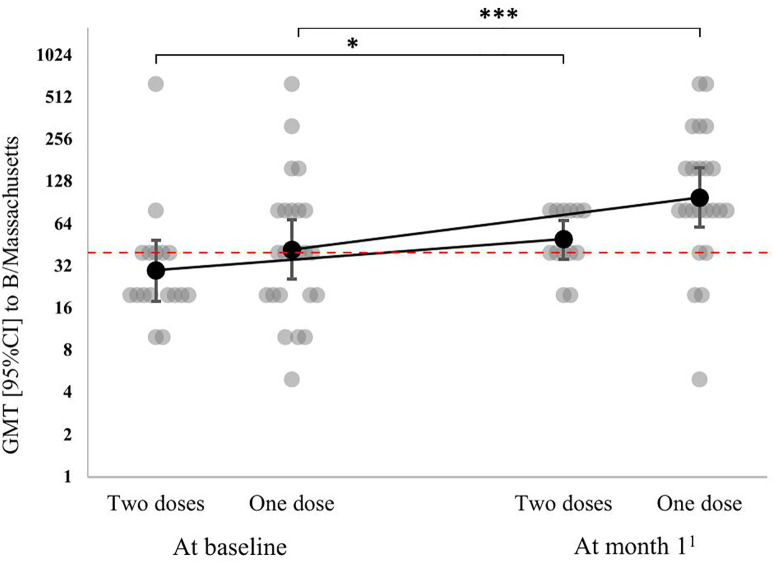
Dynamics of antibody titers to the B/Massachusetts/2/2012 strain by vaccination regimen in lung transplant candidates (individual values and GMT [95% CI]). * – p < 0.05, *** – p < 0.001 for post-hoc comparisons performed using the corresponding univariant models with a Benjamini-Yekutieli correction. ^1^ – at month 1 after the first dose in the one-dose group and at month 2 after the first dose in the two-dose group. The red broken line shows the minimal protective titer (1:40).

The antibody titers to the B/Massachusetts/2/2012 strain also depended only on the fact of vaccination (p < 0.001); the vaccination schedule did not have a statistically significant effect (p = 0.71), the dynamics of antibodies to the B/Massachusetts/2/2012 strain also did not depend on the vaccination schedule (p = 0.19) (Fig 3). At month 1, there was a rise in GMT to the B/Massachusetts/2/2012 strain from 49 [30–80] to 107 [70–172] in the one-dose group (p < 0.001) and from 35 [21–57] to 58 [40–85] in the two-dose group (p = 0.67 for comparison to the titers at month 1 after the first dose; p < 0.02 for comparison to the baseline value).

At year 1 post-vaccination, Ab titers against all the three strains returned to their baseline values regardless of the vaccination regimen (р = 0.28, р = 0.31 and р = 0.42 for comparisons with the pre-vaccination Ab titers against the A/California/7/2009(H1N1)pdm09-like, A/Texas/50/2012(H3N2) and B/Massachusetts/2/2012 strains, respectively, in the one-dose group and р = 0.06, р = 0.21 and р = 0.36, respectively, in the two-dose group) (Table 4). There were no statistically significant differences between the study groups in levels of seroprotection or Ab titers.

**Table 4:** Seroprotection levels and GMT at year 1 post-vaccination by vaccination regimen in lung transplant candidates.

Parameter	Vaccination regimen	A/California/7/2009 (H1N1)pdm09-like	A/Texas/ 50/2012(H3N2)	B/Massachusetts/ 2/2012
**Seroprotection level**	All subjects	79 [58-93]	75 [53-90]	79 [58-93]
	One-dose group	67 [30-93]% (6/9)	67 [30-93]% (6/9)	100 [66-100]% (9/9)
	Two-dose group	87 [60-98]% (13/15)	80 [52-96]% (12/15)	67 [38-88]% (10/15)
	p value	p = 0.33	p = 0.63	p = 0.12
**GMT [95% CI]**	All subjects	71 [43-118]	46 [31-69]	48 [34-66]
	One-dose group	50 [22-117]	47 [18-121]	64 [40-101]
	Two-dose group	88 [45-173]	46 [30-71]	40 [23-63]
	p value	p = 0.19	p = 0.88	p = 0.13

The seroprotection levels were analyzed using the chi-square test or Fisher’s test (with the expected rates <5%). The Ab levels were analyzed using the Mann-Whitney test.

To assess the safety of Grippol® Plus vaccine the study participants reported local and systemic adverse reactions on a daily basis over a 7-day period post-vaccination. No signs of local or systemic post-vaccination reactions were reported.

## Discussion

Immunocompromised patients should receive annual vaccinations against influenza. However, in this patient population immunological effectiveness of influenza vaccination is lower than in healthy individuals [21,22]. Patients with a long history of their conditions, individuals with frequent exacerbations of the underlying disease, and those receiving SGCs and antibacterial drugs have a poor immune response [14,23]. Thus, various strategies are being discussed to improve the immunogenicity of influenza vaccines in patients who are at high risk for severe influenza infection.

One of such strategies is the administration of two doses of an influenza vaccine during the same influenza season. The benefits of this strategy are, however, controversial. In a number of studies, cancer patients showed very low seroprotection rates 4 weeks after receiving split influenza vaccines and the use of a second vaccine dose did not improve their immune responses [24,25]. E.A. Blumberg et al. observed similar results in solid organ transplant recipients who were immunized against influenza [21]. In contrast, H. de Lavallade et al. reported a significant rise in seroprotection rates after the administration of a second influenza vaccine dose: from 39% to 68% (р = 0.008) in patients with B-cell malignancies and from 46% to 73% (р = 0.031) in allogeneic stem cell transplant recipients [26]. In another study, the administration of a second dose of an influenza vaccine was associated with a significant rise in Ab titers and an increase in the proportion of patients with a 4-fold antibody response in a group of kidney transplant recipients, which was not noted in the control group and in the group of patients with liver cirrhosis [22]. Also, the randomized controlled clinical trial TRANSGRIPE 1–2 showed that two doses of inactivated influenza vaccine given 5 weeks apart improved humoral response in solid organ transplant recipients [27].

In our study, which evaluated the immunological effectiveness of influenza vaccination in lung transplant candidates with severe bronchopulmonary disorders, the patients were immunized with an inactivated trivalent polymer-subunit influenza vaccine containing immune adjuvant (500 mcg of azoximer bromide). Azoximer bromide is used in the complex therapy of various diseases in adults and children [28–31]. Previous studies demonstrated the effectiveness and safety of this vaccine in healthy adults, pre-school and school-age children, and the elderly [32]. Other authors reported a reduction in the rates of acute respiratory infections and exacerbations of the underlying conditions as well as in the need for antibacterial drugs in patients with pulmonary disorders following vaccination with Grippol® Plus [6].

In our study seasonal influenza vaccination performed in the epidemic season preceding the study, helped maintain protective Ab titers to different influenza viral strains for a year in 46.3–58.5% of patients with bronchopulmonary disorders, regardless of their clinical status and treatment. However, of interest was whether a two-dose vaccination regimen could improve antibody response and help maintain protective Ab titers one year post-vaccination in a higher proportion of patients with severe bronchopulmonary disorders. To investigate this hypothesis, these patients were divided into two comparable groups, which were given one and two doses (one month apart) of an influenza vaccine. The two-dose regimen complied with the instructions for use of the study vaccine in certain high-risk groups, while a double-dose regimen would require additional regulatory approval for study conduct. At month 1 post-vaccination, there was a statistically significant rise in Ab titers against all the three influenza viral strains. The seroprotection rates (the percentage of subjects with a post-vaccination titer of ≥1:40), which should be > 70%, were 93 [80–98]% for the A/California/7/2009(H1N1)pdm09-like strain, 85 [70–94]% for the A/Texas/50/2012(H3N2) and 93 [80–98]% for the B/Massachusetts/2/2012 strain. Such rates are considered rather high. In patients with bronchopulmonary disorders, the one-dose regimen met the CHMP immunogenicity criteria for evaluation of influenza vaccines and was effective against all the three studied strains. In particular, it met all the criteria against the A/California/7/2009(H1N1)pdm09-like and A/Texas/50/2012(H3N2) strains and the criteria for seroprotection and seroconversion against the B/Massachusetts/2/2012 strain.

However, it is still unclear whether a second dose of influenza vaccine improves antibody response. Detailed short-term (at month 1) and long-term (at month 12) analyses of post-vaccination Ab titers did not reveal any statistically significant differences between the one-dose and two-dose groups. The administration of two vaccine doses at an interval of one month is probably justified for vaccines that include new epidemic viral strains, which have not circulated previously in human populations, and for pandemic vaccines, but these issues were beyond the scope of our investigation.

Of note, immunization with the study vaccine (an inactivated trivalent polymer-subunit influenza vaccine containing 5 mcg of each of the three influenza viral strains and 500 mcg of azoximer bromide as an immune adjuvant) and subunits vaccines, which had been used in some patients prior to inclusion in this study, yielded similar results in terms of the proportions of individuals who maintained protective Ab levels against different influenza viral strains at month 12 post-vaccination.

A disadvantage of our study is that we did not use a double dose of influenza vaccine, which would have extended our expertise, guiding us in developing strategies to maintain protective antibody titers for longer periods. Indeed, in patients with primary immunodeficiency a double-dose regimen was more beneficial than the administration of one dose of influenza vaccine [33]. Certainly, molecular and cellular mechanisms of immune response to immunization with adjuvant vaccines in patients with severe bronchopulmonary disorders remain to be investigated.

## Conclusion

Immunization of lung transplant candidates with one dose of a trivalent influenza vaccine containing 500 mcg of azoximer bromide immune adjuvant and reduced amounts of antigens (15 mcg compared to the standard amount of 45 mcg for non-adjuvanted vaccines) was effective in inducing and maintaining protective antibody titers over the corresponding influenza season.

## Supporting information

S1 File Inclusivity in global research. (DOCX)
